# Influence of OATPs on Hepatic Disposition of Erlotinib Measured With Positron Emission Tomography

**DOI:** 10.1002/cpt.888

**Published:** 2017-11-03

**Authors:** Martin Bauer, Akihiro Matsuda, Beatrix Wulkersdorfer, Cécile Philippe, Alexander Traxl, Csilla Özvegy‐Laczka, Johann Stanek, Lukas Nics, Eva‐Maria Klebermass, Stefan Poschner, Walter Jäger, Izabel Patik, Éva Bakos, Gergely Szakács, Wolfgang Wadsak, Marcus Hacker, Markus Zeitlinger, Oliver Langer

**Affiliations:** ^1^ Department of Clinical Pharmacology Medical University of Vienna Vienna Austria; ^2^ Department of Biomedical Imaging und Image‐guided Therapy, Division of Nuclear Medicine Medical University of Vienna Vienna Austria; ^3^ Center for Health & Bioresources AIT Austrian Institute of Technology GmbH Seibersdorf Austria; ^4^ Institute of Enzymology, Research Centre for Natural Sciences Hungarian Academy of Sciences Budapest Hungary; ^5^ Department of Clinical Pharmacy and Diagnostics University of Vienna Vienna Austria; ^6^ Institute of Cancer Research Medical University of Vienna Vienna Austria; ^7^ Center for Biomarker Research in Medicine CBmed GmbH Graz Austria

## Abstract

To assess the hepatic disposition of erlotinib, we performed positron emission tomography (PET) scans with [^11^C]erlotinib in healthy volunteers without and with oral pretreatment with a therapeutic erlotinib dose (300 mg). Erlotinib pretreatment significantly decreased the liver exposure to [^11^C]erlotinib with a concomitant increase in blood exposure, pointing to the involvement of a carrier‐mediated hepatic uptake mechanism. Using cell lines overexpressing human organic anion‐transporting polypeptides (OATPs) 1B1, 1B3, or 2B1, we show that [^11^C]erlotinib is selectively transported by OATP2B1. Our data suggest that at PET microdoses hepatic uptake of [^11^C]erlotinib is mediated by OATP2B1, whereas at therapeutic doses OATP2B1 transport is saturated and hepatic uptake occurs mainly by passive diffusion. We propose that [^11^C]erlotinib may be used as a hepatic OATP2B1 probe substrate and erlotinib as an OATP2B1 inhibitor in clinical drug–drug interaction studies, allowing the contribution of OATP2B1 to the hepatic uptake of drugs to be revealed.

**Study Highlights**

**WHAT IS THE CURRENT KNOWLEDGE ON THE TOPIC?**

☑ OATP2B1 is expressed in the sinusoidal membrane of hepatocytes, where it may mediate uptake of drugs from blood into liver, but its contribution to hepatic drug disposition has remained elusive due to a lack of OATP2B1‐specific probe substrates and inhibitors.

**WHAT QUESTION DID THIS STUDY ADDRESS?**

☑ We performed PET imaging with the radiolabeled tyrosine kinase inhibitor [^11^C]erlotinib and *in vitro* transport experiments to study hepatic disposition of erlotinib in humans.

**WHAT THIS STUDY ADDS TO OUR KNOWLEDGE**

☑ Our data suggest that [^11^C]erlotinib is taken up into the liver at PET microdoses by OATP2B1 without being transported by OATP1B1 and OATP1B3. Moreover, pretreatment of subjects with a therapeutic dose of erlotinib appeared to saturate OATP2B1‐mediated uptake of [^11^C]erlotinib into the liver with a concomitant increase in blood exposure.

**HOW THIS MIGHT CHANGE CLINICAL PHARMACOLOGY OR TRANSLATIONAL SCIENCE**

☑ Erlotinib may be used as an OATP2B1 inhibitor in clinical drug–drug interaction studies, allowing the contribution of OATP2B1 to the hepatic uptake of drugs to be revealed.

The liver is a major clearance organ for many drugs. There is an interplay between transporters expressed in the blood‐facing sinusoidal and bile‐facing canalicular membranes of hepatocytes and intracellular metabolizing enzymes in the hepatobiliary clearance of drugs.^1^ Uptake of drugs from blood into hepatocytes is often mediated by uptake transporters belonging to the solute carrier (SLC) family, such as organic anion‐transporting polypeptides (OATPs), whereas efflux of drugs and drug metabolites into bile is mediated by canalicular efflux transporters belonging to the adenosine triphosphate‐binding cassette (ABC) family, such as multidrug resistance‐associated protein 2 (MRP2/ABCC2). Hepatic transporters are of great relevance in clinical pharmacology, as their altered activity due to drug–drug interactions (DDIs), genetic polymorphisms, or disease may lead to pronounced changes in blood and liver exposure to drugs, which may severely impact drug safety or efficacy.^2^, ^3^, ^4^, ^5^, ^6^ In clinical studies, alterations in the activity of hepatic transporters are usually assessed by studying drug plasma pharmacokinetics. However, to better understand the impact of hepatic transporters on drug disposition, a direct assessment of drug kinetics in the liver is desirable. Noninvasive positron emission tomography (PET) imaging using ^11^C‐ or ^18^F‐labeled drugs has been proposed for the study of hepatic drug disposition and for the quantitative assessment of the effects of transporter‐mediated DDIs *in vivo*.^7^, ^8^, ^9^, ^10^ Several PET tracers have been developed as probe substrates for hepatic transporters, such as 15*R*‐[^11^C]TIC‐Me,^11^ [^11^C]dehydropravastatin,^12^ [^11^C]rosuvastatin,^13^ [^11^C]telmisartan,^14^ [^11^C]cholylsarcosine,^15^ and [^11^C]metformin.^16^ However, since many of these probes interact with several different hepatic SLC and ABC transporters, the specific contribution of individual transporters cannot be distinguished.

Erlotinib is a tyrosine kinase inhibitor (TKI) used for the treatment of nonsmall‐cell lung cancer. It is predominantly excreted via the hepatobiliary route^17^ and has been identified as a substrate of breast cancer resistance protein (BCRP/ABCG2), P‐glycoprotein (Pgp/ABCB1), organic anion transporter 3 (OAT3/SLC22A8), and organic cation transporter 2 (OCT2/SLC22A2).^18^, ^19^ It is currently not known whether hepatic uptake of erlotinib occurs via a transporter‐mediated mechanism or passive diffusion. In a preclinical PET study in mice, we observed that the rate constant for hepatic uptake of [^11^C]erlotinib was markedly (∼2‐fold) reduced in animals coinjected with a pharmacological dose of erlotinib (10 mg/kg) as compared with animals that only received a PET microdose of erlotinib, with a concomitant increase in blood exposure.^20^

In this study we assessed the hepatic disposition of erlotinib in healthy human volunteers by performing [^11^C]erlotinib PET scans without and with oral pretreatment with a therapeutic dose of erlotinib. Data were analyzed using pharmacokinetic modeling and complemented by *in vitro* experiments examining transport of [^11^C]erlotinib by different OATPs expressed in the human liver.

## RESULTS

### PET study

Study participants underwent two PET scans with [^11^C]erlotinib: a first baseline scan in which a microdose of erlotinib was administered (<10 μg), and a second scan which was performed 3 h after the oral intake of erlotinib at a dose of 300 mg, corresponding to twice a standard therapeutic dose. The study medication was well tolerated by all subjects with no occurrence of adverse events.

The percentage of radiolabeled metabolites of [^11^C]erlotinib was <10% of total radioactivity in plasma in both PET scans and at all studied timepoints. Plasma protein binding of [^11^C]erlotinib was not significantly different between scan 1 and scan 2 (scan 1: 96.9 ± 1.8%, scan 2: 95.8 ± 1.4%, *n* = 4). Pharmacokinetic parameters of unlabeled erlotinib in plasma are summarized in **Supplementary Table 1**. Maximum plasma concentration (*C*_max_) after oral intake was achieved at a median of 5 h (range 2–6 h) with a mean value of 4.6 ± 1.3 μM. Erlotinib plasma concentration at the time of the PET scan (mean of values at 3 h and 4 h after oral intake) was 4.2 ± 1.0 μM, corresponding to an unbound concentration of 0.18 μM.

**Figure 1:**
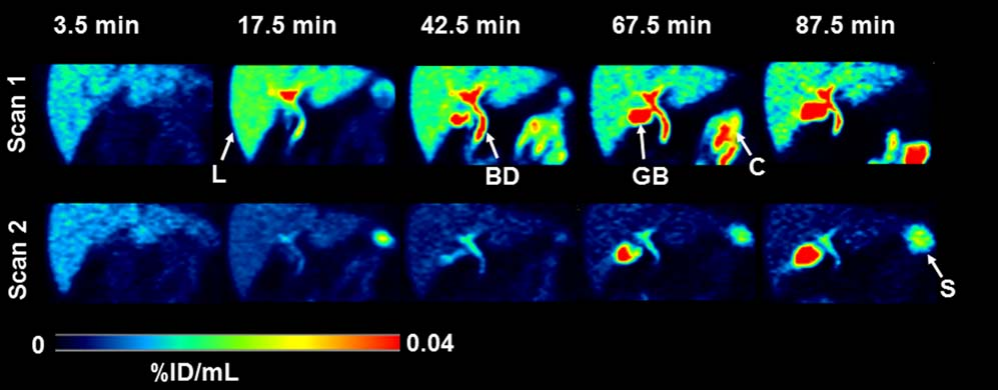
Representative PET images (subject 4) of the abdominal region for baseline scan (scan 1) and scan after oral intake of erlotinib (scan 2) recorded at 3.5, 17.5, 42.5, 67.5, and 87.5 min after radiotracer injection. Radioactivity concentration is expressed as percent of the injected dose per mL (%ID/mL) and radiation scale is set from 0 to 0.04. Anatomical structures are labeled with arrows (L, liver; GB, gall bladder; BD, bile duct; C, colon; S, spleen).

**Figure 2:**
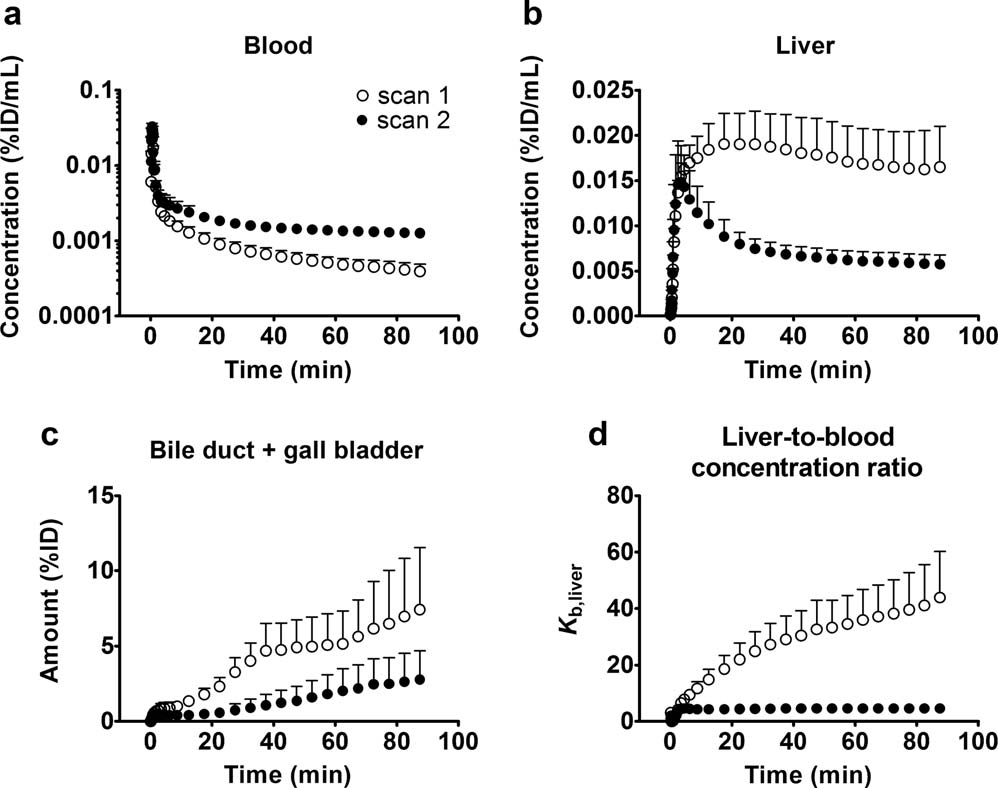
Mean time–activity curves (%ID/mL or %ID ± SD, *n* = 6) in arterial blood (**a**), liver (**b**) and bile duct and gall bladder (**c**) for baseline scan (scan 1) and scan after oral intake of erlotinib (scan 2). In **d**, the liver‐to‐blood concentration ratio (*K*_b,liver_) over time is shown for the two scans.

In **Figure** 1, representative PET images of the abdominal region are shown, indicating rapid and high radioactivity uptake in the liver and high uptake in bile duct and gall bladder at later timepoints. In scan 2, radioactivity uptake in liver, bile duct, and gall bladder was markedly reduced as compared with scan 1. In **Figure** 2, time–activity curves are shown for both scans. In scan 2, area under the curve (AUC) of [^11^C]erlotinib in blood was significantly increased as compared with scan 1 (scan 1: 0.091 ± 0.014 percent of the injected dose (%ID)/mL*min, scan 2: 0.170 ± 0.021 %ID/mL*min, change in scan 2: + 90 ± 23%). Accordingly, total clearance of [^11^C]erlotinib was significantly decreased in scan 2 (scan 1: 46 ± 10 L/h, scan 2: 10 ± 4 L/h, −77 ± 14%). While the initial uptake kinetics of radioactivity into the liver were similar in scan 1 and scan 2, the liver curves diverged at later timepoints (>4 min) in the two scans. Radioactivity continued to accumulate over time in scan 1, whereas radioactivity washed out from the liver following maximum uptake in scan 2 (**Figure** 2 **b**). The total liver exposure to radioactivity was significantly lower in scan 2 (liver AUC scan 1: 1.511 ± 0.294 %ID/mL*min, scan 2: 0.652 ± 0.096 %ID/mL*min, −56 ± 5%) and liver‐to‐blood AUC ratios of radioactivity were significantly decreased (AUC_liver_/AUC_blood_ scan 1: 17.0 ± 4.1, scan 2: 3.9 ± 0.6, −76 ± 5%). Also, liver‐to‐blood concentration ratios (*K*_*b*,liver_) were markedly lower in scan 2 (**Figure** 2 **d**). During the time course of the PET scan radioactivity was excreted into the bile duct and gall bladder, whereby the excreted radioactivity amount was significantly lower in scan 2 (bile duct and gall bladder AUC scan 1: 364 ± 126 %ID*min, scan 2: 121 ± 78 %ID*min, −67 ± 16%) (**Figure** 2 **c**). Neither in scan 1 nor in scan 2 was radioactivity excreted from the gall bladder during the time course of the PET scan (**Figure** 1).

At later timepoints of the PET scan (>40 min) some radioactivity uptake was also observed in a colon segment (left colic flexure) visible on the PET images (**Figure** 1). The concentration of radioactivity in this colon segment was significantly lower in scan 2 (colon AUC scan 1: 1.688 ± 0.442 %ID/mL*min, scan 2: 0.485 ± 0.132 %ID/mL*min, −69 ± 12%) (**Supplementary Figure 1a**). In contrast to the liver, the radioactivity concentration in the kidneys was very low and not significantly different between the two scans (**Supplementary Figure 1b**).

**Figure 3:**
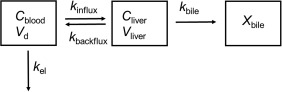
Diagram of the liver model. *C*_blood_ is the concentration of [^11^C]erlotinib in arterial blood, *C*_liver_ the concentration of [^11^C]erlotinib in the liver, and *X*_bile_ the total amount of [^11^C]erlotinib in the bile duct and gall bladder. *V*_d_ and *V*_liver_ denote volume of distribution and physiological liver volume, respectively. *K*_influx_ (min^−1^) is the influx rate constant from blood into liver, *k*_backflux_ (min^−1^) the backflux rate constant from liver into blood, *k*_bile_ (min^−1^) the rate constant for biliary excretion, and *k*_el_ (min^−1^) the rate constant for elimination of [^11^C]erlotinib from blood. *K*_influx_/*k*_backflux_ defines the apparent [^11^C]erlotinib influx into the liver at steady state. Hepatic uptake clearance (*CL*_uptake_, mL min^−1^) is defined as *k*_*influx*_ × *V*_*d*_.

**Figure 4:**
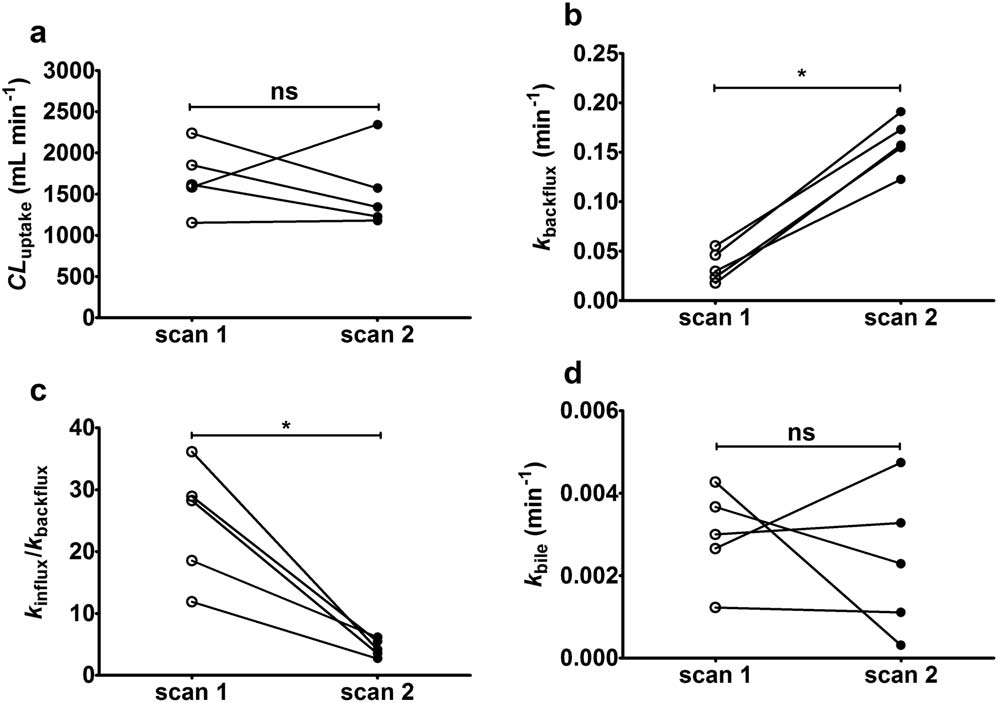
*CL*_uptake_ (**a**), *k*_backflux_ (**b**), *k*_influx_/*k*_backflux_ (**c**), and *k*_bile_ (**d**) in individual subjects for baseline scan (scan 1) and scan after oral intake of erlotinib (scan 2). ns, not significant, *P < 0.05, Wilcoxon matched‐pairs signed rank test.

PET data were analyzed using pharmacokinetic modeling to estimate the influx rate constant from blood into liver (*k*_influx_), hepatic uptake clearance (*CL*_uptake_), the backflux rate constant from liver into blood (*k*_backflux_), and the rate constants for biliary excretion (*k*_bile_) and for elimination of [^11^C]erlotinib from blood (*k*_el_) (**Figure** 3). In addition, the apparent [^11^C]erlotinib influx into the liver at steady state (*k*_influx_/*k*_backflux_) was calculated. The model did not provide physiologically meaningful results for Subject 5, in that *CL*_uptake_ greatly exceeded the liver blood flow, so that this subject was excluded from the modeling analysis. Fitted curves for one representative subject are shown in **Supplementary Figure 2.** In **Table** 1, modeling outcome parameters are given for five subjects and in **Figure** 4 changes in outcome parameters are depicted for individual subjects. In scan 2, *k*_backflux_ was significantly increased (scan 1: 0.035 ± 0.016 min^−1^, scan 2: 0.160 ± 0.025 min^−1^, + 435 ± 232%) and *k*_influx_/*k*_backflux_ was significantly decreased (scan 1: 24.7 ± 9.5, scan 2: 4.4 ± 1.4, −80 ± 9%). In addition, there was a trend for decreases in *k*_influx_, *CL*_uptake_, *k*_bile_, and *k*_el_ in scan 2 (*k*_influx_ scan 1: 0.74 ± 0.11 min^−1^, scan 2: 0.70 ± 0.28 min^−1^; *CL*_uptake_ scan 1: 1,687 ± 397 mL min^−1^, scan 2: 1,532 ± 478 mL min^−1^; *k*_bile_ scan 1: 0.0030 ± 0.0012 min^−1^, scan 2: 0.0023 ± 0.0018 min^−1^; *k*_el_ scan 1: 0.069 ± 0.057 min^−1^, scan 2: 0.063 ± 0.040 min^−1^), but these differences were not statistically significant.

**Table 1:** Outcome parameters from liver model for baseline scan and scan after oral intake of erlotinib in individual subjects^a^

Subject	Baseline				After oral erlotinib			*k*_el_ (min^−1^)
	*k*_influx_ (min^−1^) *CL*_uptake_ (mL min^−1^) ^*b*^	*k*_backflux_ (min^−1^)	*k*_bile_ (min^−1^)	*k*_el_ (min^−1^)	*k*_influx_ (min^−1^) *CL*_uptake_ (mL min^−1^) ^*b*^	*k*_backflux_ (min^−1^)	*k*_bile_ (min^−1^)	
1	0.86 ± 0.09 2,236	0.030 ± 0.010	0.0027 ± 0.0000	0.021 ± 0.074	0.67 ± 0.09 1,572	0.122 ± 0.027	0.0047 ± 0.0005	0.031 ± 0.014
2	0.66 ± 0.08 1,851	0.055 ± 0.013	0.0012 ± 0.0002	0.141 ± 0.037	0.46 ± 0.10 1,342	0.173 ± 0.052	0.0011 ± 0.0001	0.036 ± 0.011
3	0.64 ± 0.07 1,152	0.018 ± 0.005	0.0030 ± 0.0000	0.022 ± 0.062	0.65 ± 0.10 1,178	0.157 ± 0.034	0.0033 ± 0.0003	0.060 ± 0.013
4	0.67 ± 0.06 1,610	0.024 ± 0.004	0.0037 ± 0.0000	0.120 ± 0.061	0.54 ± 0.09 1,227	0.155 ± 0.038	0.0023 ± 0.0003	0.059 ± 0.014
6	0.85 ± 0.07 1,585	0.046 ± 0.009	0.0043 ± 0.0004	0.041 ± 0.036	1.17 ± 0.17 2,343	0.191 ± 0.040	0.0003 ± 0.0000	0.131 ± 0.026

Parameter estimates are given as mean ± standard deviation. *k*_influx_, influx rate constant from blood into liver, *k*_backflux_, backflux rate constant from liver into blood, *k*_bile_, rate constant for biliary excretion, *k*_el_, elimination rate constant from blood, *CL*_uptake_, hepatic uptake clearance.

a Data for subject 5 are not reported as the model did not provide physiologically meaningful results.

Calculated by multiplying *k*_influx_ with volume of distribution (*V*_d_).

### *In vitro* uptake experiments

**Figure 5:**
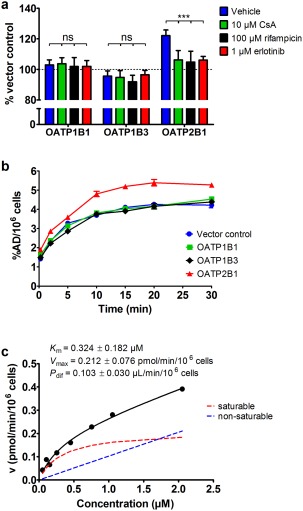
(**a**) Mean uptake of [^11^C]erlotinib (<0.1 μM) after 5‐min incubation time (% of vector control ± SD) in OATP1B1, OATP1B3, and OATP2B1 overexpressing cells treated with vehicle, cyclosporine A (CsA, 10 μM), rifampicin (100 μM), or unlabeled erlotinib (1 μM). (**b**) Time dependency of [^11^C]erlotinib (<0.1 μM) uptake (percent of applied dose per 10^6^ cells, %AD/10^6^ cells ± SD) in cells overexpressing OATP1B1, OATP1B3, or OATP2B1 or transfected with the empty vector (vector control). (**c**) Concentration dependency of the OATP2B1‐specific uptake of [^11^C]erlotinib determined at 7.5 min at various erlotinib concentrations (0.05–2.05 μM). Black solid line represents model fit and broken red and blue lines represent fits for saturable and nonsaturable components, respectively. Kinetic parameters ± standard error are shown in the graph. Details of the fitting are described in the **Supplementary Methods**. Data shown in **a** are from two experiments performed with three technical replicates each and data shown in **b** and **c** are from one experiment performed with three technical replicates each. ns, not significant, ***P < 0.001, two‐way ANOVA with Bonferroni posttest.

To investigate if [^11^C]erlotinib is a substrate of OATPs expressed in the liver, we performed *in vitro* uptake experiments using tracer concentrations (<0.1 μM) of [^11^C]erlotinib in A431 cells engineered to stably overexpress OATP1B1 (SLCO1B1), OATP1B3 (SLCO1B3), or OATP2B1 (SLCO2B1) (**Figure** 5). After 5 min incubation time at pH 7.4, uptake of [^11^C]erlotinib was not significantly different between cells transfected with the empty vector and cells overexpressing OATP1B1 or OATP1B3. In contrast, in OATP2B1 overexpressing cells, [^11^C]erlotinib uptake was significantly higher (+22%). We incubated cells with [^11^C]erlotinib in the presence of an excess of unlabeled erlotinib (1 μM) or the prototypical OATP inhibitors cyclosporine A (10 μM) or rifampicin (100 μM). All three compounds significantly decreased [^11^C]erlotinib uptake in OATP2B1 overexpressing cells to comparable levels as in vector control cells, while the same treatment had no effect in OATP1B1 and OATP1B3 overexpressing cells (**Figure** 5 **a**). We also assessed the time dependency of [^11^C]erlotinib uptake into OATP‐overexpressing and vector control cells, which indicated higher uptake of [^11^C]erlotinib in OATP2B1‐overexpressing cells as compared with the other cells, with a maximum difference reached after 10–15 min of incubation time (**Figure** 5 **b**). Kinetic analysis revealed both a saturable and nonsaturable component for OATP2B1‐specific uptake of [^11^C]erlotinib (**Figure** 5 **c**). The Michaelis constant (*K*_m_) of the saturable component was 0.324 ± 0.182 μM.

## DISCUSSION

In this study we used PET imaging to assess hepatic disposition of [^11^C]erlotinib and found a pronounced decrease in liver uptake when subjects were pretreated with an oral therapeutic dose of erlotinib. This pointed to the involvement of a carrier‐mediated hepatic uptake mechanism of erlotinib. Although erlotinib is a frequently used drug, the mechanism of its liver uptake has not yet been identified. Erlotinib was found not to be transported by the major hepatic uptake transporters OATP1B1 and OATP1B3.^21^ In addition, it has been shown that erlotinib is not a substrate of OCT1 (SLC22A1) and OAT2 (SLC22A7), two other uptake transporters expressed in hepatocytes.^19^ Erlotinib was found to be a very potent inhibitor of OATP2B1.^22^, ^23^ Johnston *et al*. reported a half‐maximum inhibitory concentration (IC_50_) of 0.03 μM of erlotinib for OATP2B1 inhibition and a 3,467‐ and 40‐fold lower potency for inhibition of OATP1B1 (IC_50_: 104 μM) and OATP1B3 (IC_50_: 1.19 μM), respectively.^23^ It was suggested that erlotinib is a competitive inhibitor of OATP2B1,^23^ but its transport by OATP2B1 has not been directly assessed so far. Sato *et al*. found that the structurally related TKI gefitinib was transported by OATP2B1.^24^ We hypothesized that the reduction in [^11^C]erlotinib liver uptake observed in our study following pretreatment with erlotinib may be due to saturation of [^11^C]erlotinib transport by OATP2B1. We performed *in vitro* uptake experiments with tracer concentrations (<0.1 μM) of [^11^C]erlotinib in cell lines overexpressing different OATP isoforms (**Figure** 5 **a**). We found a significantly higher uptake of [^11^C]erlotinib as compared to control cells in OATP2B1 overexpressing cells, and a reduction in cellular uptake when unlabeled erlotinib or the prototypical OATP inhibitors cyclosporine A or rifampicin were added. Kinetic experiments indicated that OATP2B1 is a high‐affinity and low‐capacity transporter of erlotinib (**Figure** 5 **c**). Our data confirm the findings by Khurana *et al*. that erlotinib is not transported by OATP1B1 and 31B3.^21^ For the first time, we show that erlotinib at low concentrations is transported by OATP2B1 and that transport is saturated at therapeutic erlotinib concentrations. The relatively small difference in [^11^C]erlotinib uptake between control and OATP2B1 overexpressing cells suggests that cellular uptake of [^11^C]erlotinib is not exclusively transporter‐dependent, but also occurs via passive diffusion, which is supported by the high lipophilicity of erlotinib (logP: 4.19).^23^

*In vivo*, [^11^C]erlotinib was rapidly taken up into the liver after intravenous (i.v.) injection followed by sustained liver uptake for the duration of the PET scan. Oral pretreatment with erlotinib markedly reduced liver uptake of [^11^C]erlotinib with a concomitant decrease in total clearance of [^11^C]erlotinib. This dose dependency in [^11^C]erlotinib clearance is in good agreement with a dose escalation study of i.v. erlotinib in cancer patients, in whom a decrease in clearance was observed with increasing erlotinib dose.^25^ The mean unbound plasma concentration of unlabeled erlotinib at the time of the PET scan (0.18 μM) was in a similar range as the *in vitro K*_m_ value for erlotinib transport by OATP2B1 (0.32 μM). As there is evidence that portal blood concentrations of drugs after oral dosing may be several times higher than peripheral blood concentrations,^26^ it appears possible that the reduced liver distribution of [^11^C]erlotinib after oral erlotinib was caused by saturation of OATP2B1‐mediated liver uptake of [^11^C]erlotinib.

PET data were analyzed using a pharmacokinetic model (**Figure** 3), yielding *k*_influx_, *CL*_uptake_, *k*_backflux_, and *k*_bile_ as outcome parameters for the liver. In scan 1, *CL*_uptake_ was 21 ± 4 mL min^−1^ kg^−1^, which was close to the hepatic blood flow (21 mL min^−1^ kg^−1^),^27^ and therefore corresponded to a hepatic extraction ratio close to 1. Contrary to what would be expected from the inhibition of an uptake transporter^11^, ^12^, ^14^ and contrary to our preclinical results,^20^ *CL*_uptake_ was only moderately reduced in some subjects in scan 2 (**Figure** 4 **a**). The liver time–activity curves showed very similar initial hepatic uptake kinetics in the two PET scans (**Figure** 2 **b**). This agrees quite well with the *in vitro* uptake kinetics (**Figure** 5 **b**), in which the maximum difference between OATP2B1‐overexpressing and vector control cells was reached at later timepoints. [^11^C]Erlotinib rapidly washed out from the liver in scan 2 at timepoints >4 min, whereas it continued to accumulate in the liver in scan 1 (**Figure** 2 **b**). This behavior was reflected by a pronounced increase (+435%) in *k*_backflux_ in scan 2 (**Figure** 4 **b**), which may have masked a decrease in *CL*_uptake_. Interestingly, a similar phenomenon has been observed in a study by Pfeifer *et al*., who assessed the effect of the OATP inhibitor ritonavir on liver distribution of the single‐photon emission computed tomography (SPECT) tracer [^99m^Tc]mebrofenin, which is a substrate of OATP1B1, OATP1B3, MRP2, and MRP3.^28^ Those authors reported a 46% reduction in *CL*_uptake_ and a 517% increase in the efflux clearance of [^99m^Tc]mebrofenin from liver to blood following ritonavir pretreatment. Pfeifer *et al*. attributed this effect to an induction of sinusoidal efflux transporters (MRP3/ABCC3, MRP4/ABCC4) by ritonavir and/or displacement of protein binding of [^99m^Tc]mebrofenin in the liver. Erlotinib was found not to be transported by MRP4,^19^ and it is currently not known whether it is a substrate of MRP3. We hypothesize that the increase in *k*_backflux_ in our study may have been related to saturation of OATP2B1 transport activity. In scan 2, unlabeled erlotinib may have displaced protein binding of [^11^C]erlotinib in the liver, leading to an increase in the hepatic unbound fraction, which could have driven passive back‐diffusion of [^11^C]erlotinib across the basolateral membrane from liver into blood. Saturation of OATP2B1 by unlabeled erlotinib may have prevented OATP2B1‐mediated reuptake of [^11^C]erlotinib from blood into liver, which may have contributed to the increase in *k*_backflux_. This is supported by a significant positive correlation between the percent change in *k*_backflux_ in scan 2 and the plasma AUC of unlabeled erlotinib from time zero to the end of the PET scan (Spearman correlation coefficient *r* = 1.00, *P* = 0.017, data not shown). This hypothesis remains to be proven in future experiments. It is noteworthy that in another clinical PET study, which assessed the effect of a hepatic uptake transporter (OCT1) on liver distribution of a drug ([^11^C]metformin), also an increase in *k*_backflux_ was found under conditions when uptake transport was impaired (i.e., in carriers of genetic *SLC22A1* polymorphisms).^16^ To reflect changes in both *k*_influx_ and *k*_backflux_ in scan 2, we calculated *k*_influx_/*k*_backflux_ as the apparent [^11^C]erlotinib influx into the liver at steady state, which was significantly decreased (by 80%) in scan 2 (**Figure** 4 **c**). Our assumption that liver uptake in scan 2 occurs by passive diffusion is supported by the observation that liver and blood time–activity curves were parallel in scan 2, which was not the case in scan 1 (**Supplementary Figure 3**). The rate constant for transport of [^11^C]erlotinib from hepatocytes into bile, *k*_bile_, was reduced in three out of five subjects following pretreatment with erlotinib (**Figure** 4 **d**). This is in good agreement with our preclinical data in mice, which showed a 5‐fold reduction in *k*_bile_ of [^11^C]erlotinib when unlabeled erlotinib was coinjected, presumably due to saturation of Bcrp and Pgp transport.^20^

The liver receives dual blood supply via the hepatic artery (∼25%) and via the portal vein (∼75%). Blood from the portal vein cannot be sampled in humans, and our attempts to generate an image‐derived portal input function were unsuccessful. We therefore did not consider tracer delivery via the portal vein in our pharmacokinetic model, which may have led to an underestimation of modeling outcome parameters^29^ but should not have affected relative changes in outcome parameters between scans.

Over the time course of the PET scan [^11^C]erlotinib may also undergo direct intestinal secretion from blood, as reflected by delayed radioactivity uptake in the colon (**Figure** 1). This process may be transporter‐mediated, because a decrease in the concentration of radioactivity secreted into colon was observed after pretreatment with erlotinib (**Supplementary Figure 1a**). A compartment for intestinal excretion of radiotracer was not included in our pharmacokinetic model, as only a small colon segment was within the field of view of the PET scanner and we were not able to determine the total amount of radioactivity excreted into the intestine. Renal uptake of [^11^C]erlotinib was negligible, which is in accordance with low renal excretion of erlotinib.^17^ Erlotinib is predominately excreted in the form of metabolites into feces, whereas in plasma unchanged erlotinib represents the major circulating compound.^17^ In agreement with literature data, we found only a low amount of radiolabeled metabolites of [^11^C]erlotinib in plasma. In an earlier study in mice, we measured the content of radiolabeled metabolites of [^11^C]erlotinib in different tissues.^20^ We found that the majority of radioactivity in plasma, liver, and bile was in the form of unchanged [^11^C]erlotinib, suggesting that metabolism of [^11^C]erlotinib may not be as important over the short duration of a PET scan.

OATP‐mediated DDIs are of great concern in drug development, and current guidelines by the European Medicines Agency and the US Food and Drug Administration recommend *in vitro* studies to assess the interaction of new drug candidates with OATP1B1 and OATP1B3. In selected cases in which an OATP‐mediated DDI risk cannot be excluded, clinical studies employing prototypical OATP inhibitors (e.g., rifampicin or cyclosporine A) or nonselective OATP probe substrates (e.g., rosuvastatin, pravastatin, pitavastatin) are recommended.^30^ Whereas the role of OATP1B1 and 1B3 in hepatic uptake of drugs is firmly established, the contribution of OATP2B1 to hepatic drug disposition has so far remained elusive.^2^ This may be related to the fact that most drugs that show affinity for OATP2B1 are also transported by OATP1B1 and/or 1B3 and that OATP2B1‐selective inhibitors for *in vivo* use are not available.^31^ Although certain drugs inhibit OATP2B1 (e.g., gemfibrozil, cyclosporine A),^32^ clinically relevant DDIs that can be specifically attributed to hepatic OATP2B1 have not yet been reported.

Our data suggest that erlotinib is taken up into the human liver at PET microdoses both by OATP2B1 and by passive diffusion, whereas at therapeutic doses erlotinib transport by OATP2B1 is saturated so that liver uptake occurs mainly by passive diffusion. We propose that [^11^C]erlotinib may be used as a hepatic OATP2B1 probe substrate and erlotinib as an OATP2B1 inhibitor in clinical liver DDI studies. OATP2B1 inhibition with erlotinib may help reveal the contribution of OATP2B1 to the hepatic uptake of drugs which are transported by several OATPs expressed in the liver. On the other hand, caution is warranted when combining erlotinib with OATP2B1 substrates (e.g., statins) in the clinic, as this may lead to clinically relevant DDIs due to OATP2B1 inhibition in the liver and rises in blood concentrations of victim drugs. One study evaluated the antitumor activity of a combination of erlotinib with the OATP2B1 substrate rosuvastatin^32^ in 24 patients with advanced solid malignancies.^33^ In this study, muscle toxicity was observed at a substantially higher rate (34%) than during standard statin therapy (1–5%), with seven cases of myalgia and one case of rhabdomyolysis resulting in a study‐related death. Muscle toxicity of rosuvastatin may have been caused by inhibition of OATP2B1‐mediated hepatic rosuvastatin uptake by erlotinib, leading to increases in rosuvastatin blood exposure.

In conclusion, despite the fact that previous studies have indicated that erlotinib interacts with multiple SLC and ABC transporters, our data suggest that PET with [^11^C]erlotinib can measure OATP2B1 transport activity in the human liver, suggesting a potential utility as a hepatic OATP2B1 probe substrate for *in vivo* DDI studies. Unexpectedly, uptake transporter inhibition was mainly associated with an increase in *k*_backflux_ rather than a decrease in *CL*_uptake_ in our pharmacokinetic model. As the contribution of OATP2B1 to the liver uptake of [^11^C]erlotinib appeared to be quite small, an OATP2B1‐specific probe substrate with lower passive permeability than erlotinib may be preferable for future studies. Our study is a unique example of a drug which shows transporter‐mediated liver uptake at PET microdoses but not at therapeutic doses, leading to nonlinear pharmacokinetics.

## METHODS

The study was registered under EUDRACT number 2015‐001593‐18, approved by the Ethics Committee of the Medical University of Vienna and conducted in accordance with the Declaration of Helsinki and its amendments. Written consent was obtained from all subjects. Study participants were defined as healthy based on medical history, physical examination, and routine blood and urine laboratory testing. Further, subjects were required to be free of any medication for at least 14 days before start of the study, which was confirmed by a urine drug screen.

### Radiotracer synthesis

[^11^C]Erlotinib was synthesized following a previously published procedure^34^ and formulated in sterile phosphate‐buffered saline solution containing 8.6% (v/v) ethanol. Molar radioactivity at the time of injection was 61 ± 51 GBq/μmol (*n* = 12 batches) and radiochemical purity was >98%.

### Imaging and sampling

One female and five male healthy volunteers (mean age: 27 ± 6 years, mean weight: 79 ± 7 kg) were enrolled in the study. All subjects underwent magnetic resonance imaging (MRI) of the upper abdomen (T1‐ and T2‐weighted MAGNETOM Skyra 3.0T MRI, Siemens Medical Solutions, Erlangen, Germany) and two dynamic [^11^C]erlotinib PET scans conducted on separate days on an Advance scanner (General Electric Medical Systems, Milwaukee, WI). [^11^C]Erlotinib was injected as an i.v. bolus over 20 sec (injected radioactivity: 365 ± 20 MBq, corresponding to 5 ± 4 μg of unlabeled erlotinib). Radioactivity in the upper abdominal region was measured over 90 min by employing a consecutive frame sequence of 1 × 15, 3 × 5, 3 × 10, 2 × 30, 3 × 60, 2 × 150, and 16 × 300 sec. In parallel to PET imaging, serial blood samples were drawn from the radial artery for the first 2.5 min, followed by samples at 3.5, 5, 10, 20, 30, 40, 60, and 80 min after radiotracer injection. For both PET scans, subjects were asked to fast for at least 6 h before radiotracer injection. Approximately 3 h before start of the second PET scan, an oral dose of 300 mg erlotinib (Tarceva, Roche Pharma, Nutley, NJ; two 150‐mg tablets) was administered. Ten blood samples were collected at baseline and hourly for 8 h and at ∼21 h after erlotinib intake. Blood samples were centrifuged to obtain plasma, which was kept at −80°C until analysis of erlotinib concentrations.

### Blood and metabolite analysis

Aliquots of blood and plasma were measured for radioactivity in a gamma‐counter, which was cross‐calibrated with the PET camera. Selected plasma samples were analyzed for radiolabeled metabolites of [^11^C]erlotinib (**Supplementary Methods**). Due to the low amount of radiolabeled metabolites in plasma, total radioactivity counts were used for construction of an arterial input function. Plasma protein binding of [^11^C]erlotinib was determined by incubating a plasma sample obtained immediately before each PET scan with [^11^C]erlotinib for 20 min at 37°C, followed by ultrafiltration using Microcon‐10 kDa centrifugal filter units with an Ultracel‐10 membrane (Millipore, Bedford, MA). The concentration of unlabeled erlotinib in plasma was determined by high‐performance liquid chromatography with ultraviolet detection (**Supplementary Methods**). Pharmacokinetic parameters of erlotinib in plasma were determined with the Kinetica 2000 software package, v. 3.0 (InnaPhase).

### Imaging data analysis

Regions of interest (ROIs) for liver, combined bile duct and gall bladder, left renal cortex and left colic flexure were manually delineated on individual MRI data coregistered to the respective PET data using PMOD 3.6 (PMOD Technologies, Zurich, Switzerland). Radioactivity in the bile duct and gall bladder was assumed to correspond to radioactivity excreted into bile. Radioactivity concentrations in tissue (kBq/mL) were expressed as percent of the injected dose per mL (%ID/mL) or in case of bile duct and gall bladder as %ID (by multiplication with the total ROI volume, see **Supplementary Table 2**) vs. time.

### Modeling

Assuming that only the systemic blood, liver, bile duct, and gall bladder need to be considered as distribution organs, that excretion from bile does not occur during the PET study and that measured radioactivity in tissue corresponds to unmetabolized [^11^C]erlotinib, a compartment model for liver distribution was constructed (**Figure** 3). Hepatic tracer delivery via the portal vein was not considered in the model. The following differential equations were used:

(1) $$V_{d}\frac{dC_{\text{blood}}}{dt}=k_{\text{backflux}}\times C_{\text{liver}}\times V_{\text{liver}}-k_{\text{influx}}\times C_{\text{blood}}\times V_{d}-k_{\text{el}}\times C_{\text{blood}}\times V_{d}$$

(2) $$V_{\text{liver}}\frac{dC_{\text{liver}}}{dt}=k_{\text{influx}}\times C_{\text{blood}}\times V_{d}-k_{\text{backflux}}\times C_{\text{liver}}\times V_{\text{liver}}-k_{\text{bile}}\times C_{\text{liver}}\times V_{\text{liver}}$$

(3) $$\frac{dX_{\text{bile}}}{dt}=k_{\text{bile}}\times C_{\text{liver}}\times V_{\text{liver}}$$

where *V*_d_ and *V*_liver_ (mL) are the volume of distribution and physiological liver volume, respectively (**Supplementary Table 3**). *C*_blood_ and *C*_liver_ (kBq/mL) are the blood and liver concentrations of [^11^C]erlotinib, respectively. *X*_bile_ (kBq) is the total amount of [^11^C]erlotinib in bile duct and gall bladder. *K*_influx_, *k*_backflux_, and *k*_bile_ (min^−1^) are the rate constants for radiotracer transfer between blood, liver, and bile and *k*_el_(min^−1^) is the rate constant for elimination of [^11^C]erlotinib from blood (**Figure** 3). *V*_d_ was estimated by fitting a two‐compartment model to the blood concentration profiles of [^11^C]erlotinib. *V*_liver_ was calculated by a previously reported method.^35^ Blood concentration was obtained from [^11^C]erlotinib plasma concentration and the plasma‐to‐blood concentration ratio. *C*_liver_ was calculated from the PET data using:

(4) $$C_{\text{liver}}=\frac{C_{\text{PET}}-0.20\times C_{\text{blood}}}{0.80}$$

where *C*_PET_ is the liver concentration including the concentration in the vascular space in the liver.^36^ *K*_influx_, *k*_backflux_, *k*_bile_, and *k*_el_ were estimated by fitting the data using the multir program.^37^ Hepatic uptake clearance (*CL*_uptake_, mL min^−1^) was calculated as *k*_*influx*_ × *V*_*d*_.

### Cell lines and *in vitro* uptake experiments

Different *in vitro* uptake experiments were performed with [^11^C]erlotinib in human epidermoid carcinoma A431 cells expressing different OATPs (OATP1B1, OATP1B3, or OATP2B1) and in A431 cells transfected with the empty vector, which had been generated as described elsewhere (Patik *et al*., submitted), as outlined in the **Supplementary Methods**.

### Statistical analysis

All values are given as mean ± standard deviation (SD). Statistical testing was performed using Prism 6.0 software (GraphPad Software, San Diego, CA). Differences in outcome parameters between scans were tested using the Wilcoxon matched‐pairs signed rank test. For analysis of *in vitro* data, differences between groups were analyzed by two‐way analysis of variance followed by a Bonferroni posttest. The level of statistical significance was set to a *P* value of less than 0.05.

## CONFLICT OF INTEREST

The authors declare that they have no conflicts of interest.

## AUTHOR CONTRIBUTIONS

O.L., M.B., A.M., and G.S. wrote the article; O.L., M.B., A.M., C.Ö‐L., M.H., and M.Z. designed the research; M.B., B.W., C.P., A.T., L.N., E‐M.K., C.Ö‐L., J.S., S.P., I.P., É.B., and W.W. performed the research; M.B., A.M., A.T., C.Ö‐L., J.S., W.J., and O.L. analyzed the data; C.Ö‐L., I.P., É.B., and G.S. contributed new reagents/analytical tools.

## Supporting information

Supporting Information 1

Supporting Information 2

Supporting Information 3

Supporting Information 4

Supporting Information 5

Supporting Information 6

Supporting Information 7

Supporting Information 8
