# Supplementary material for: Influence of OATPs on Hepatic Disposition of Erlotinib Measured With Positron Emission Tomography
Source: Clin Pharmacol Ther. 2017 Nov 3;104(1):139–47. doi: 10.1002/cpt.888 (PMC6083370; doi:10.1002/cpt.888)
Supplement: Supplementary file 1 — Supporting Information 1 [file CPT-104-139-s001.docx]

**Supplementary Methods**

**Analysis of radiolabeled metabolites of [^11^C]erlotinib in plasma**

Plasma samples collected at 3.5, 5, 10, 20, 30, 40 and 60 min were analyzed for radiolabeled metabolites of [^11^C]erlotinib using a previously described solid-phase extraction assay.[^1^](#_ENREF_1) In brief, plasma (2-4 mL) was diluted with water (2 mL) containing 80 µL 5M aqueous hydrochloric acid and loaded on a Sep-Pak tC2 cartridge (Waters Corp.), which had been pre-activated with methanol (3 mL) and water (5 mL). The cartridge was first washed with water (5 mL) and then eluted with methanol (3 mL). Radioactivity in all three fractions (plasma, water and methanol) was measured in a gamma-counter. Radioactivity in the plasma and water fractions contained polar radiolabeled metabolites of [^11^C]erlotinib, whereas unchanged [^11^C]erlotinib was recovered in the methanol fraction.

**Determination of unlabeled erlotinib in plasma**

The concentration of unlabeled erlotinib in plasma was determined by high-performance liquid chromatography (HPLC) using a Dionex UltiMate 3000 system (Dionex Corp., Sunnyvale, USA) with ultraviolet (UV) detection at 331 nm. After the addition of 600 µL ice-cold methanol to 200 µL of plasma, the samples were centrifuged (13,000 g for 5 min) and 80 µL of clear supernatant was injected onto a Hypersil BDS-C18 column (5 µm, 250 x 4.6 mm I.D., Thermo Fisher Scientific, Inc, Waltham, USA), preceded by a Hypersil BDS-C18 pre-column (5 µm, 10 x 4.6 mm I.D.) and kept at 40°C. The mobile phase consisted of a continuous gradient mixed from aqueous ammonium acetate buffer (10 mM, pH = 5.0) (solvent A) and acetonitrile (solvent B) at a flow rate of 1.0 mL/min. Solvent B linearly increased from 30% (0 min) to 60% at 9 min, increased further to 90% at 9.5 min, and was kept constant at 90% until 14.5 min. Linear calibration curves were generated by spiking drug-free human plasma with standard solutions of erlotinib to obtain a concentration range of 0.01 to 5.00 µg/mL (average correlation coefficients > 0.999). For this method, the limit of quantification for erlotinib was determined to be 10 ng/mL in human plasma (coefficients of accuracy and precision were < 8%).

***In* *vitro* uptake experiments**

Human epidermoid carcinoma A431 cells overexpressing different OATPs (OATP1B1, OATP1B3 or OATP2B1) were generated as described elsewhere (Patik *et al.*, submitted). OATP overexpression was confirmed by Western blotting. Functionality of the OATPs was verified by [^3^H]estrone-3-sulfate uptake experiments. A431 cells transfected with the empty vector were used as a negative control. All cell lines were cultured in Dulbecco's modified eagle medium supplemented with 10% fetal bovine serum, 2 mM L-glutamine, 100 units/mL penicillin and 100 µg/mL streptomycin. Cells were maintained at 37°C in a humidified atmosphere of 95% air and 5% CO_2_. For the uptake experiments, 5 x 10^5^ cells per well were seeded in 24-well tissue culture plates (VWR, Radno, PA, USA) and incubated for 24 h. On the day of the experiment, growth medium was aspired and cells were washed with pre-warmed transport buffer (Hank's balanced salt solution supplemented with 10 mM *N*-2-hydroxyethylpiperazine-*N*-2-ethane sulfonic acid). For inhibition experiments, pre-warmed transport buffer containing either unlabeled erlotinib (1 µM), cyclosporine A (10 µM), rifampicin (100 µM) or DMSO (1%, control) was added and cells were incubated for 30 min. Then the drug solutions were removed and cells were washed with pre-warmed transport buffer. Then pre-warmed transport buffer supplemented with 0.002% (v/v) polysorbate-80 (to prevent non-specific binding of [^11^C]erlotinib) and containing [^11^C]erlotinib (~ 2 MBq per well, 0.06 ± 0.02 µM) and either the inhibitors or DMSO at the same concentrations as stated above was added and cells were incubated for another 5 min. For time dependency experiments, pre-warmed transport buffer was added and cells were incubated for 30 min. Then the transport buffer was removed and pre-warmed transport buffer supplemented with 0.002% (v/v) polysorbate-80 and containing [^11^C]erlotinib (~ 2 MBq per well, 0.05 µM) was added and cells were incubated for additional 0.3, 2, 5, 10, 15, 20 and 30 min. For concentration dependency experiments, pre-warmed transport buffer was added and cells were incubated for 30 min. Then the transport buffer was removed and pre-warmed transport buffer supplemented with 0.002% (v/v) polysorbate-80 and containing [^11^C]erlotinib (~ 2 MBq per well, 0.05 µM) and either unlabeled erlotinib (50, 100, 200, 400, 700, 1,000 and 2,000 nM) or DMSO (1%, control) was added and cells were incubated for additional 7.5 min. In all experiments, cell count was determined on separate 24-well tissue culture plates before or after each experiment. In all experiments, radiotracer solutions were aspired following incubation and cells were washed twice with ice-cold transport buffer. Cells were detached using trypsin-EDTA (0.25%) and transport buffer was added to transfer the cell suspension into tubes, which were measured for radioactivity in a gamma-counter. Each inhibition experiment was performed 2 times with 3 technical replicates each and radioactivity in cells was corrected for radioactive decay, normalized to 10^6^ cells and expressed as percent of vector control (% control). The time dependency experiment was performed once with 3 technical replicates and radioactivity in cells was corrected for radioactive decay and expressed as percent of applied dose per 10^6^ cells (%AD/10^6^ cells). The concentration dependency experiment was performed once with 3 technical replicates and radioactivity in cells was corrected for radioactive decay. Kinetic parameters were determined using a previously described method.[^2^](#_ENREF_2) In brief, cellular radioactivity uptake was converted into units of pmol/min/10^6^ cells. OATP2B1-specific uptake of erlotinib was determined by subtracting the uptake into cells transfected with the empty vector from the uptake into cells transfected with OATP2B1. The following equation was used to obtain kinetic parameters:

$v=\frac{V_{\max}\times S}{K_{m}+S}+P_{\mathrm{dif}}\times S$ (1)

where *v* is the uptake velocity of erlotinib (pmol/min/10^6^ cells), *S* is the erlotinib concentration in the transport buffer (µM), *K*_m_ is the Michaelis constant (µM), *V*_max_ is the maximum uptake velocity (pmol/min/10^6^ cells), and *P*_dif_ is the non-saturable uptake clearance (µL/min/10^6^ cells). Curve fitting was performed by the non-linear regression method with a modified Michaelis-Menten model according to equation 1 using Prism 6.0 software (GraphPad Software).

**Supplementary references**

1. Bahce, I. *et al.* Development of [^11^C]erlotinib positron emission tomography for in vivo evaluation of EGF receptor mutational status. *Clin. Cancer Res.* **19**, 183-93 (2013).

2. Hirano, M., Maeda, K., Shitara, Y. & Sugiyama, Y. Contribution of OATP2 (OATP1B1) and OATP8 (OATP1B3) to the hepatic uptake of pitavastatin in humans. *J. Pharmacol. Exp. Ther.* **311**, 139-46 (2004).
