# Supplementary material for: Influence of OATPs on Hepatic Disposition of Erlotinib Measured With Positron Emission Tomography
Source: Clin Pharmacol Ther. 2017 Nov 3;104(1):139–47. doi: 10.1002/cpt.888 (PMC6083370; doi:10.1002/cpt.888)
Supplement: Supplementary file 2 — Supporting Information 2 [file CPT-104-139-s002.docx]

**Supplementary Table 1 Pharmacokinetic data of unlabeled erlotinib in plasma**

| Subject | *C*_max_ (µM) | *T*_max_ (h) | *C*_PET_ (µM) | *C*_PET,unbound_ (µM) | *AUC*_0-∞_ (µM*h) | *T*_1/2_ (h) |
| --- | --- | --- | --- | --- | --- | --- |
| 1 | 3.51 | 2 | 3.30, 3.33 | n.d. | 68.1 | 14.1 |
| 2 | 5.03 | 6 | 4.12, 4.50 | 0.12, 0.13 | 94.0 | 12.5 |
| 3 | 4.74 | 4 | 4.73, 4.74 | n.d. | 72.0 | 9.2 |
| 4 | 6.43 | 6 | 4.91, 5.63 | 0.20, 0.23 | 102.8 | 11.3 |
| 5 | 2.83 | 6 | 2.66, 2.71 | 0.10, 0.10 | 36.6 | 5.4 |
| 6 | 4.92 | 4 | 4.61, 4.92 | 0.28, 0.30 | 187.8 | 28.7 |

*C*_max_, maximum plasma concentration, *T*_max_, time of maximum plasma concentration, *C*_PET_, plasma concentrations at time of the PET scan (i.e. at 3 h and 4 h after oral dosing), *C*_PET,unbound_, unbound plasma concentrations at time of the PET scan, *AUC*_0-∞_, area under the curve from time zero to infinity, *T*_1/2_, terminal elimination half-life, n.d., not determined
