# Supplementary material for: Influence of OATPs on Hepatic Disposition of Erlotinib Measured With Positron Emission Tomography
Source: Clin Pharmacol Ther. 2017 Nov 3;104(1):139–47. doi: 10.1002/cpt.888 (PMC6083370; doi:10.1002/cpt.888)
Supplement: Supplementary file 3 — Supporting Information 3 [file CPT-104-139-s003.docx]

**Supplementary Table 2 Volumes of interest (cm^3^) used in PET data analysis**

|  | Scan 1 | Scan 2 |  |
| --- | --- | --- | --- |
| Liver | 1,257 ± 128 | 1,070 ± 67 |  |
| Bile duct + gall bladder | 92 ± 10 | 82 ± 29 |  |
| Kidney cortex | 23 ± 7 | 27 ± 17 |  |
| Colon | 57 ± 24 | 73 ± 73 |  |

Stated are mean values ± standard deviation
