# Supplementary material for: Influence of OATPs on Hepatic Disposition of Erlotinib Measured With Positron Emission Tomography
Source: Clin Pharmacol Ther. 2017 Nov 3;104(1):139–47. doi: 10.1002/cpt.888 (PMC6083370; doi:10.1002/cpt.888)
Supplement: Supplementary file 4 — Supporting Information 4 [file CPT-104-139-s004.docx]

**Supplementary Table 3 Physiological parameters used in the liver model**

|  | *V*_d_ (mL) | |  |
| --- | --- | --- | --- |
| Subject | Scan 1 | Scan 2 | *V*_liver_ (mL) |
| 1 | 2,596 | 2,349 | 1,851 |
| 2 | 2,819 | 2,890 | 1,870 |
| 3 | 1,792 | 1,804 | 1,580 |
| 4 | 2,416 | 2,252 | 1,533 |
| 6 | 1,856 | 1,999 | 1,678 |

*V*_d_*,* volume of distribution

*V*_liver_*,* physiological liver volume
