# Supplementary material for: Influence of OATPs on Hepatic Disposition of Erlotinib Measured With Positron Emission Tomography
Source: Clin Pharmacol Ther. 2017 Nov 3;104(1):139–47. doi: 10.1002/cpt.888 (PMC6083370; doi:10.1002/cpt.888)
Supplement: Supplementary file 8 — Supporting Information 8 [file CPT-104-139-s008.doc]

**Supplementary Methods**. Data shown in **a** are from 2 experiments performed with 3 technical replicates each and data shown in **b** and **c** are from 1 experiment performed with 3 technical replicates each. ns, not significant, ****p* < 0.001, two-way ANOVA with Bonferroni post-test.

**Supplementary Data**

**Supplementary Methods**

**Supplementary Table 1** Pharmacokinetic data of unlabeled erlotinib in plasma.

**Supplementary Table 2** Volumes of interest (cm^3^) used in PET data analysis.

**Supplementary Table 3** Physiological parameters used in the liver model.

**Supplementary Figure 1** Mean time-activity curves (%ID/mL ± SD, *n* = 6) in a colon segment (left colic flexure) (**a**) and in kidney cortex (**b**) for baseline scan (scan 1) and scan after oral intake of erlotinib (scan 2).

**Supplementary Figure 2** Observed and fitted time-activity curves of [^11^C]erlotinib in one representative subject (subject 4) in arterial blood (**a**), liver (**b**) and bile duct and gall bladder (**c**). Data is expressed in the same units as used for modeling (kBq/mL or kBq). Observed liver concentrations shown in **b** are liver concentrations measured with PET minus radioactivity concentrations in arterial blood scaled to 20% (see **Methods**). Initial radioactivity uptake observed in the bile duct and gall bladder (**c**) is most likely related to spill in of radioactivity from the liver at early time points.

**Supplementary Figure 3** Mean time-activity curves (%ID/mL ± SD, *n* = 6) for liver and arterial blood for baseline scan (scan 1, **a**) and scan after oral intake of erlotinib (scan 2, **b**).
